# IUC26694-92 Stereotactic Body Radiotherapy and Immunotherapy for Metastatic Renal Cell Carcinoma: Croatian Uro-Oncology Network Experience

**DOI:** 10.1093/oncolo/oyag286.028

**Published:** 2026-08-21

**Authors:** H Brcic, J Murgic, K Brcic, M Lekic, I Tomaskovic, M Jazvic, D Schwarz, M Penc, T Omrcen, A Frobe

**Affiliations:** Sestre milosrdnice University Hospital Centre, Department of Oncology and Nuclear Medicine, Zagreb - Croatia; Sestre milosrdnice University Hospital Centre, Department of Oncology and Nuclear Medicine, Zagreb - Croatia; Sestre milosrdnice University Hospital Centre, Department of Haematology, Zagreb - Croatia; Special Hospital Radiochirurgia Zagreb, Sveta Nedjelja - Croatia; Sestre milosrdnice University Hospital Centre, Department of Urology, Zagreb - Croatia; Sestre milosrdnice University Hospital Centre, Department of Oncology and Nuclear Medicine, Zagreb - Croatia; Special Hospital Radiochirurgia Zagreb, Sveta Nedjelja - Croatia; University Hospital Centre Osijek, Department of Oncology, Osijek - Croatia; University Hospital Centre Split, Department of Oncology and Radiotherapy, Split - Croatia; Sestre milosrdnice University Hospital Centre, Department of Oncology and Nuclear Medicine, Zagreb - Croatia

## Abstract

**Background:**

Stereotactic body radiotherapy (SBRT) is increasingly used for metastasis-directed treatment in metastatic renal cell carcinoma (mRCC) treated with immune checkpoint inhibitors (ICI). We evaluated outcomes according to SBRT timing relative to ICI, accounting for immortal-time bias.

**Methods:**

This retrospective real-world cohort, from the Croatian Uro-Oncology Network, included 204 patients with mRCC treated with ipilimumab/nivolumab or nivolumab in four Croatian centres. Patients were classified as no SBRT, pre-ICI SBRT, on-ICI SBRT, or post-ICI SBRT. Baseline differences were assessed using nonparametric and categorical tests. Progression-free survival (PFS) and overall survival (OS) were estimated from ICI start and, among SBRT-treated patients, from the first SBRT. On-ICI SBRT was modelled as a time-dependent exposure in Cox regression.

**Results:**

Fifty-three patients (26.0%) received SBRT: 23 (43.4%) pre-ICI, 21 (39.6%) on-ICI, and 9 (17.0%) post-ICI; 151 (74.0%) did not receive SBRT. Median follow-up, by reversed Kaplan-Meier, was 44.7 months. Groups differed by age, ECOG performance status, IMDC risk, brain metastases, oligometastatic status, and nephrectomy. From ICI start, median PFS was 19.1, 15.0, 35.5, and 68.8 months for no SBRT, pre-ICI, on-ICI, and post-ICI SBRT, respectively (log-rank p  =  0.070). Corresponding median OS was 29.4, 32.8, 67.1, and 93.7 months (log-rank p  =  0.005). In adjusted time-dependent Cox analysis (n  =  190), on-ICI SBRT was not independently associated with PFS (hazard ratio [HR] 1.29, 95% confidence interval [CI] 0.63-2.67) or OS (HR 0.68, 95% CI 0.29-1.61). Sensitivity analysis restricted to no SBRT versus on-ICI SBRT was consistent for PFS (HR 1.30, 95% CI 0.67-2.50) and OS (HR 0.68, 95% CI 0.31-1.51). From the first SBRT, median PFS was 29.3, 17.7, and 21.4 months, and median OS was 40.3, 54.0, and 42.9 months for pre-, on-, and post-ICI SBRT, respectively.

**Conclusions:**

SBRT-treated patients had favourable descriptive outcomes, particularly when SBRT occurred during or after ICI. However, baseline imbalances and time-dependent modelling indicate that the apparent Kaplan-Meier advantage is not sufficient evidence of an independent survival benefit. These data support SBRT as a feasible metastasis-directed strategy in selected mRCC patients and justify prospective evaluation.

